# Cationic Residues of the HIV-1 Nucleocapsid Protein Enable DNA Condensation to Maintain Viral Core Particle Stability during Reverse Transcription

**DOI:** 10.3390/v16060872

**Published:** 2024-05-29

**Authors:** Helena Gien, Michael Morse, Micah J. McCauley, Ioulia Rouzina, Robert J. Gorelick, Mark C. Williams

**Affiliations:** 1 Department of Physics, Northeastern University, Boston, MA 02115, USA; h.gien@northeastern.edu (H.G.); mi.morse@northeastern.edu (M.M.); m.mccauley@northeastern.edu (M.J.M.); 2 Department of Chemistry and Biochemistry, Center for Retroviral Research and Center for RNA Biology, The Ohio State University, Columbus, OH 43210, USA; irouzina@gmail.com; 3 AIDS and Cancer Virus Program, Frederick National Laboratory for Cancer Research, Frederick, MD 21702, USA; gorelicr@mail.nih.gov

**Keywords:** DNA condensation, optical tweezers, atomic force microscopy, capsid uncoating, HIV-1 nucleocapsid protein

## Abstract

The HIV-1 nucleocapsid protein (NC) is a multifunctional viral protein necessary for HIV-1 replication. Recent studies have demonstrated that reverse transcription (RT) completes in the intact viral capsid, and the timing of RT and uncoating are correlated. How the small viral core stably contains the ~10 kbp double stranded (ds) DNA product of RT, and the role of NC in this process, are not well understood. We showed previously that NC binds and saturates dsDNA in a non-specific electrostatic binding mode that triggers uniform DNA self-attraction, condensing dsDNA into a tight globule against extending forces up to 10 pN. In this study, we use optical tweezers and atomic force microscopy to characterize the role of NC’s basic residues in dsDNA condensation. Basic residue mutations of NC lead to defective interaction with the dsDNA substrate, with the constant force plateau condensation observed with wild-type (WT) NC missing or diminished. These results suggest that NC’s high positive charge is essential to its dsDNA condensing activity, and electrostatic interactions involving NC’s basic residues are responsible in large part for the conformation, size, and stability of the dsDNA-protein complex inside the viral core. We observe DNA re-solubilization and charge reversal in the presence of excess NC, consistent with the electrostatic nature of NC-induced DNA condensation. Previous studies of HIV-1 replication in the presence of the same cationic residue mutations in NC showed significant defects in both single- and multiple-round viral infectivity. Although NC participates in many stages of viral replication, our results are consistent with the hypothesis that cationic residue mutations inhibit genomic DNA condensation, resulting in increased premature capsid uncoating and contributing to viral replication defects.

## 1. Introduction

The mature HIV-1 capsid is a sub-viral particle released into the cytoplasm of infected cells. The capsid is a cone-shaped structure formed by ~1200 spontaneously assembling capsid proteins (CA), which organize in a primarily hexameric lattice. Exactly twelve pentameric lattice units are incorporated per capsid, allowing the capsid to achieve its closed shape [1,2,3,4]. Capsids can vary in size, but typically measure ~120 nm in length and ~60 nm in diameter [5,6]. The 9.8 kbp-long viral genomic RNA (gRNA) dimer is co-packaged in the small intra capsid space along with $\sim 2.4\times 10^{3}$ nucleocapsid (NC) proteins, ~200 molecules of the viral replication enzymes reverse transcriptase and integrase, and a few other viral and host proteins necessary for reverse transcription (RT) and other steps further downstream in the viral life cycle. The RT process produces a double-stranded DNA (dsDNA) copy of the viral gRNA, which is eventually integrated into the host chromosome. The integrated provirus is transcribed into viral mRNA and then translated by the host cell machinery into viral proteins, which can form new infectious viral particles.

For many years, it was believed the capsid would disassemble, or “uncoat”, either immediately after entry into the cytoplasm, gradually while in transit to the nuclear envelope, or while docking at a nuclear pore [7,8,9,10,11]. An uncoating event occurring outside the nucleus was thought to be necessitated by the incompatibility between the capsid diameter (~60 nm), then presumed to be rigid, and the diameter of the nuclear pore (~30 nm) [12]. These early uncoating models assumed the RT complex (RTC) could support RT while partially or fully exposed to the cell cytoplasm. However, the cytosolic environment has proven hostile to the RTC; cytoplasmic DNA intermediates can trigger an innate cell immune response, leading to pyroptotic cell death and abortive HIV-1 infection [13,14]. In addition to protecting the core contents from their external environment, the intact capsid is critical in keeping replication enzymes associated with the RTC until RT has progressed to completion [11], and is likely required for the capsid contents to enter the cell nucleus by import through a nuclear pore [15,16,17,18,19]. Recent studies have suggested a new order of post-entry events consistent with these capsid functions, with intact capsids persisting beyond nuclear import and uncoating near the integration site <1.5 h before integration [20,21,22,23]. Observed uncoating occurs abruptly, within three minutes of initial capsid rupture and approximately two hours after the completion of reverse transcription [20,22]. Further studies have confirmed that an endogenous reverse transcription (ERT) process can complete within intact cores in vitro and in the nucleus [21,24,25]. The progression of ERT was observed to lead to the rupture of the core in both atomic force microscopy (AFM) and cryo-EM studies [26,27]. AFM studies following the rigidity and shape of intact cores during the progression of ERT revealed a series of spikes in capsid stiffness and an overall non-uniform capsid swelling, which could be eliminated by RT inhibitors. RT inhibitors also strongly delayed capsid uncoating, directly linking the progression of ERT with forcible capsid disassembly [9,28].

Estimates show [29] that prior to ERT, the inner capsid volume is ~50–70% occupied by the viral gRNA dimer and its co-packaged viral proteins. It remains unclear how the other 30–50% of available capsid volume can stably contain the full-length ~10 kbp dsDNA product of ERT. In our previous work [30], we found NC binds dsDNA and strongly condenses it into a tight globule, leading to a significant reduction in the expected net capsid content volume. However, we expect that as the viral RNA dimer template is reverse transcribed into proviral dsDNA, the total amount of nucleic acid in the capsid grows and eventually doubles, while the amount of NC within the capsid remains constant. While gRNA and transiently created single-stranded (ss) DNA, are not as reliant on NC for compaction, due to their flexible ss backbone, they effectively compete with dsDNA for NC binding [29]. As such, the NC present in the capsid is likely insufficient to stably condense the entire full length reverse transcribed viral dsDNA. In cell measurements, it was confirmed that increased viral gRNA length and the completion of reverse transcription are required for efficient uncoating [29].

**Figure 1:**
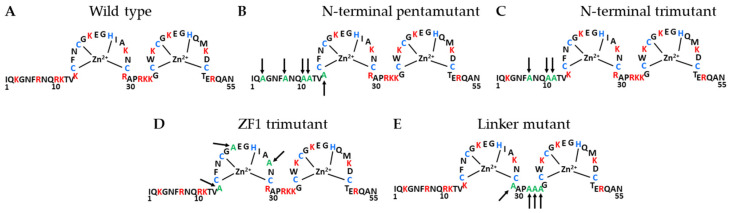
Sequence of wild-type (WT) and basic residue mutation variants of the HIV-1 nucleocapsid protein (NC). Zinc binding residues are highlighted in blue and basic residues are highlighted in red. Individual mutated residues are shown in green and indicated with an arrow. (**A**) WT; (**B**) K3A/R7A/R10A/K11A/K14A (N-terminal pentamutant); (**C**) R7A/R10A/K11A (N-terminal trimutant); (**D**) K14A/K20A/K26A (zinc finger 1 trimutant); (**E**) R29A/R32A/K33A/K34A (zinc finger linker mutant). The sequence shown is for the NL4-3 isolate (GenBank accession no. AF324493).

Our previous findings [30] suggest HIV-1 NC is an extremely efficient dsDNA condensing agent that induces a strong uniform DNA self-attraction. This condensing activity may be analogous to dsDNA collapse by small multivalent cations with a charge ≥+3 [31,32,33,34]. NC is a 55 amino acid-long, highly basic protein with 15 positive and 4 negative residues which contribute to its +11 net charge. However, salt dependence measurements of NC-nucleic acid (NA) binding [35,36] show NC to have an effective charge of only ~+3.5. NC’s comparatively small effective charge implies not all its positively charged residues are in close contact with dsDNA phosphates upon binding [37], possibly due to some of NC’s cationic residues being positioned within its rigid CCHC zinc coordinating domains, or zinc fingers (ZF) (Figure 1). Besides the dominant electrostatic interactions between NC’s cationic residues and NA phosphate groups, NC’s two aromatic residues (one in each of its two zinc fingers) are known to stack with unpaired bases, specifically with guanine [38,39,40]. This leads to a modest specificity in NC binding some RNA structures containing unpaired bases, specifically guanines [35,41,42], such as the Psi region at the 5’end of viral RNA that plays a role in its selective packaging into new virions [40,43]. NC’s ability to stack its two aromatic residues with unpaired NA bases is known to be responsible for NC’s well-documented NA-chaperoning activity. NA-chaperoning by the NC domain of Gag is required for genomic RNA (gRNA) packaging and primer placement, and chaperoning by mature NC plays a critical role in the maturation of the gRNA dimer and the specificity of reverse transcription [38]. However, such non-electrostatic NC contacts are expected to be minimal for a molecule with no unpaired bases, as is the case for a dsDNA molecule under physiological force conditions. We further confirmed that NC condenses multiple sequences, including HIV-1 DNA and multiple plasmids, to the same degree [29]. As expected for the primarily electrostatic nature of NC-dsDNA binding, we find the majority of NC dissociates from dsDNA within 1 min [30]. The high mobility of NC bound to the dsRNA TAR hairpin has also been characterized [44,45]. Consequently, we expect NC to condense dsDNA via a primarily electrostatic counterion correlation mechanism [46,47]. If this is the case, NC’s cationic residues should play a major role in its ability to condense dsDNA. We also tested the counterion correlation mechanism prediction that excessive amounts of multivalent cation binding to dsDNA will dissolve condensed dsDNA back into solution. This is accompanied by a complex charge reversal [47,48,49], or “overcharging”, which has been observed for dsDNA condensed by small multivalent cations such as Spermine^+4^ (Spe^+4^), Spermidine^+3^ (Spd^+3^), Cobalt Hexamine^+3^ (CoHex^+3^) [50,51], polysulphanates [52], histone1 [53], and protamine [53,54,55,56]. In this study, we make use of several NC variants with subsets of cationic residues mutated to neutral alanine (Figure 1). Compared to wild-type (WT) NC, we observe a drastic and varying reduction in these variants’ ability to bind and condense dsDNA, suggesting cationic residues contribute differentially to dsDNA condensation depending on their location in NC’s structure. The mutations probed here were previously shown to lead to defects in HIV-1 replication [57], suggesting an inability to effectively condense DNA and stabilize viral core particles may be partly responsible for previously observed defects in HIV-1 replication.

## 2. Materials and Methods

### 2.1. Protein Purification and DNA Substrate

Recombinant WT NL4-3 HIV-1 NCp7, 55-amino acid protein was produced and purified as described previously [58,59]. Recombinant HIV-1 NC mutants were produced and purified as described previously [57]. Mutations are as follows: K3A/R7A/R10A/K11A/K14A (N-terminal pentamutant); R7A/R10A/K11A (N-terminal trimutant); K14A/K20A/K26A (zinc finger 1 trimutant); (E) R29A/R32A/K33A/K34A (zinc finger linker mutant). All mutations were verified by NA sequence analysis. WT and mutant amino acid sequences are presented in Figure 1. DNA substrates were labeled as previously described [60]. Transfer plasmid pBACguss-11 (gift from Borja Ibarra, IMDEA Nanociencia) was double digested with restriction enzymes BamHI and SacI (New England Biolabs, NEB, Ipswich, MA, USA). Biotinylated oligos (Integrated DNA Technologies, Coralville, IA, USA) were ligated to both resulting overhangs using T4 DNA ligase (NEB). The sample was then gel purified to remove excess biotinylated oligos and DNA concatemers.

### 2.2. Optical Tweezers

Force spectroscopy measurements were taken on a custom-built optical tweezers (OT) instrument [61,62,63]. In each experiment, a double-biotinylated DNA (8.1 kbp) substrate is tethered between two small (1.7 µm) streptavidin-coated micro-beads. One bead is held in a counterpropagating dual-beam laser trap, while the other is manipulated by a mobile micropipette tip. The pipette is moved by a piezoelectric stage, which controls the DNA end-to-extension to within 0.1 nm precision. The deflection of the trapping laser is recorded and used to calculate the tension applied across the DNA. Bright field images are taken and used to calculate a force drift correction associated with each data point. The sample chamber is connected to multiple inlet tubes, which exchange the solution conditions surrounding the DNA molecule. Experiments are performed in a 50 mM Na^+^ buffer solution (10 mM HEPES, 45 mM NaCl, 5 mM NaOH, pH 7.5), unless otherwise specified. All experiments were performed with ≥3 replicates (each using a new DNA substrate) to account for the stochastic nature of this system and to establish mean and standard error values. Error bars in plots represent standard error unless otherwise noted.

**Figure 2:**
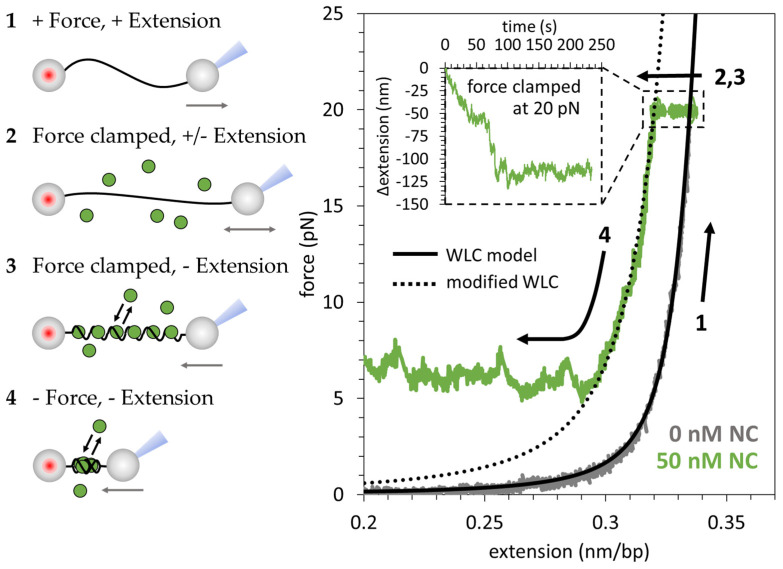
NC’s electrostatic binding mode collapses dsDNA into a dense globule along a constant force plateau. (**1**) Protein-free double-stranded (ds)DNA (gray) is extended until reaching 20 pN substrate tension, following the extensible worm-like chain (WLC) model (black line). The force-extension profile for bare dsDNA is consistent with the WLC polymer model. (**2**) A force feedback loop is applied to clamp the construct tension at 20 pN while 50 nM HIV-1 nucleocapsid protein (NC) is flowed into the cell. (**3**) NC binds the dsDNA (green). The NC-dsDNA complex reaches an equilibrium state within ~150 s. (**4**) The force clamp is removed, and the end-to-end distance is reduced in a controlled manner. At first, the force extension profile is consistent with a modified WLC model with a shortened persistence length (dotted line). Below a critical force, the force-extension profile deviates from the modified WLC model. In this low-force regime, NC maintains an approximately constant tension across the substrate as the end-to-end distance is reduced.

dsDNA is a long, semiflexible, anionic polymer. In the absence of dsDNA binding proteins, its force-extension behavior is well-described by the extensible worm-like chain (WLC) model (Figure 2, black line) with a contour length of b=0.34 nm/bp, persistence length p=45 nm and elastic stretch modulus s=1200 pN, in agreement with previous measurements [64]. The extensible WLC model gives the following expression for the protein-free dsDNA end-to-end extension x as a function of the applied force F:

(1) $$x\left(F\right)=b\left(1-\frac{1}{2}{\left(\frac{k_{b}T}{p\cdot F}\right)}^{\frac{1}{2}}+\frac{F}{s}\right)$$

To probe the interaction between NC and dsDNA, a single 8.1 kbp dsDNA molecule is isolated with an optical tweezers instrument. Once isolated, the dsDNA is extended to apply 20 pN of tension (Figure 2(1)). The force-extension profile observed upon stretching is consistent with the WLC model described above. Agreement of the data with the model verifies that the fundamental polymer properties p, b, and s remain unchanged in this force-extension regime. A force-feedback loop is applied to clamp the system at 20 pN (extension is automatically increased or decreased to maintain tension) while NC is flowed into the sample (Figure 2(2)). After equilibration of the dsDNA length at a fixed 20 pN force (Figure 2(3)), the force clamp is removed, and the extension is reduced (Figure 2(4)). Initially, the force-extension profile is consistent with a modified WLC model with a shortened persistence length of ~20 nm [30]. Note that the exact persistence length is difficult to measure, as proper fitting for OT data is most sensitive the low force regime where NC condenses dsDNA and proper fitting for AFM data requires precise tracing of the dsDNA backbone, which is obscured by condensation and dsDNA intersections. Instead, the effective persistence length after NC binding is estimated based on the reduction in dsDNA elongation at high force using OT and end to end displacement for dsDNA deposited on AFM surface. Below a critical force, the data deviate from the modified WLC and a constant force plateau is observed. This plateau corresponds to the force with which slack is incorporated into the NC-dsDNA complex, resulting in constant tension across the substrate, even at low extensions. See our previous publication [30] for a more detailed examination of the force-extension behavior of wild-type NC. In this work, the force-extension behavior of cationic NC variants is compared to the characteristic force-extension profile for the wild-type, shown in detail in Figure 2.

### 2.3. AFM Imaging

Phage M13mp18 (NEB) was linearized by BamHI digestion and diluted to concentration of 100 pM in a buffer containing 150 mM NaCl, 10 mM Hepes (pH 7.5), and 10 μM spermidine. The sample was incubated with fixed concentrations of NC, either WT or mutant, for 1 min before 5 μL of sample was deposited on a freshly cleaved mica surface. The surface was then rinsed with excess DI water and then blown dry under a stream of argon gas. The sample was imaged using a MultiMode 8 AFM and Nanoscope V controller (Bruker, Billerica, MA, USA) in peak force tapping mode. Data were analyzed and images were exported using Gwyddion software (version 2.66).

## 3. Results

### 3.1. Force-Extension Measurements and AFM Imaging Show NC Softens, Condenses, Overcharges and Re-Solubilizes dsDNA

**Figure 3:**
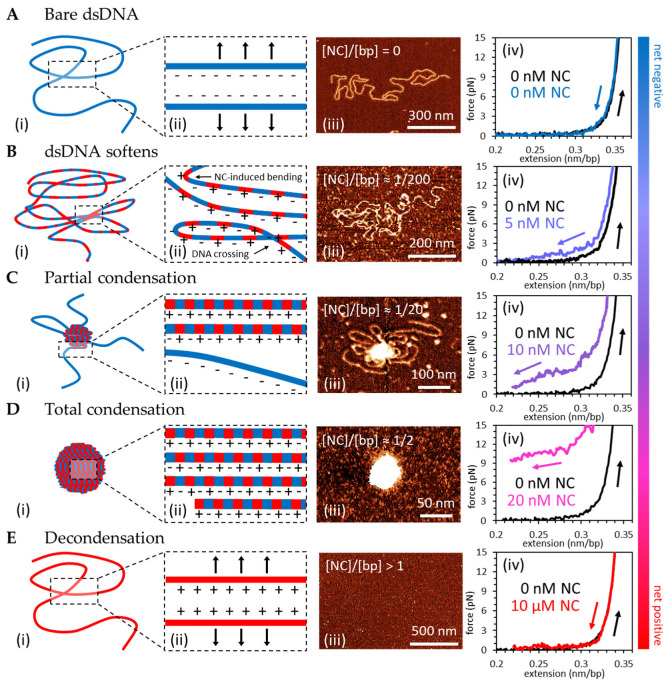
NC-induced overcharging of dsDNA. (**A**) Bare double-stranded (ds)DNA has a large persistence length (~50 nm) limiting DNA crossings and increasing end-to-end distance (i) due to dsDNA’s uniform negative charge preventing self-interaction (ii). Atomic force microscopy (AFM) imaging (iii) and force-extension measurements (iv) are consistent with the extensible worm-like chain (WLC) model with a persistence and contour length typical for B-DNA. (**B**) Low, sub-saturating concentrations of HIV-1 nucleocapsid protein (NC) effectively soften (reduce the rigidity of) dsDNA by inducing local bending. AFM imaging shows increased flexibility and frequent dsDNA intersections while force-extension curves lack the typical condensation force plateau (iv). The overall charge of the complex is negative, as the net charge of bound NC does not exceed the dsDNA net charge. (**C**) AFM images show a partial condensate surrounded by single layer dsDNA while the force-extension curves exhibit a partial condensation force plateau that eventually decays to zero force due to uncondensed DNA. The probability of phase separation occurring increases with increasing NC:bp. (**D**) Stoichiometrically optimized NC:bp binding (one NC per ~5 bp [30]) leads to complete dsDNA condensation into the tight globule. Mutual repulsion between mobile dsDNA-bound NC proteins might create a periodic +/− charge pattern on the dsDNA surface that arranges in counterphase (+ vs. −) against an analogous +/− charge pattern on an adjacent dsDNA, thereby leading to the mutual attraction schematically depicted in (**D**(ii)). A maximum density NC/dsDNA globule is observed on AFM and force-extension curve observes complete NC/dsDNA condensation along maximum condensation force plateau ($F_{c}=\sim 9\;pN$). (**E**) Oversaturating dsDNA with NC leads to the inversion of the complex net charge from negative to positive and de-condensation. Charge inversion prevents attachment to the positively charged AFM surface and a condensation force plateau and dsDNA softening are absent in the force-extension curves.

As proviral dsDNA is synthesized during ERT, the total amount of nucleic acid inside the capsid doubles [29] while the amount of NC remains constant. Therefore, we are interested in the effect of varying NC/dsDNA saturation levels on NC-induced dsDNA compaction. In our previous work [30], we explored the relationship between [NC] and the strength of the condensation force. We observed that in 50 mM Na^+^, the condensation force appears at ~10 nM NC, reaches a maximum of 9 +/− 1 pN at 20 nM NC, and tends to decrease beyond 50 nM NC. Here, we show that at yet higher [NC], the condensation force decreases to ~5 pN at 1 µM NC and completely vanishes at 10 µM NC. In this concentration regime, we observe NC-induced dsDNA self-attraction to be eliminated, indicating dsDNA re-solubilization at the bulk ([NC] > ~10 µM). We observe the same trends by visualizing NC/dsDNA complexes with atomic force microscopy (AFM) (Figure 3). Note that higher concentrations of NC are used in AFM experiments for three technical reasons. First, there is a fixed ratio of DNA to protein in the bulk incubation, such that free NC is exhausted as it binds the DNA, unlike the OT experiments where NC is effectively unlimited. Second, the incubation step is limited to only 1 min, as multiple DNA molecules start to aggregate, in contrast to OT experiments where a single DNA molecule is isolated. Finally, the attachment of negatively charged DNA to the negatively charged mica surface is mediated through the trivalent cation spermidine [65], which effectively competes with NC for binding to DNA. Additionally, both OT and AFM experiments replicate capsid like conditions in one important aspect each. OT experiments isolate a single dsDNA molecule, like in the capsid, which is suspended in a large sample chamber that contains an effectively inexhaustible supply of NC, unlike the capsid. As such, the dsDNA can be fully condensed with low bulk concentrations of NC. In contrast, AFM experiments use a fixed volume solution of dsDNA and NC, which conserves total protein number as in the capsid, such that high initial NC concentration is required to fully condense the dsDNA as free protein concentration drastically reduces during binding. However, to avoid interactions between separate dsDNA molecules (aggregation), which do not occur in the capsid, the dsDNA must be dilute and the incubation time short, unlike the small capsid volume, which keeps both local NA and NC concentrations high. Thus, neither experiment fully replicates capsid-like conditions, but the combination of conditions tested can be used to approximate these conditions.

Our OT and AFM results are summarized in Figure 3, with increasing [NC] conditions arranged from top to bottom. For each concentration regime, the four columns in Figure 3 depict: (i) a schematic of the conformation of the NC/dsDNA complex; (ii) the details of the charge distribution within the NC/dsDNA complex, based on the counterion correlation mechanism [46,47]; (iii) a representative AFM scan; and (iv) a representative force-extension profile. In the absence of NC, the dsDNA substrate is rigid, negatively charged, and its force-extension profile is consistent with the WLC model for unperturbed B-DNA (Figure 3A). NC concentrations leading to low levels of NC/dsDNA saturation (Figure 3B) do not produce dsDNA condensation—this is reported by both the absence of the condensation force plateau and by the uncondensed NC/dsDNA globules observed with AFM. While this low level of NC saturation does not induce dsDNA condensation, it does have a dsDNA “softening” effect, which is apparent in both the reduction of DNA rigidity in AFM images (Figure 3B(iii)), and by the shortened persistence length obtained by fitting force-extension data to the WLC model (Equation (1)) (Figure 3B(iv)). In the range of concentrations studied in this work, the persistence length of the complex varies from ~50 nm (no NC bound) to ~20 nm (saturated NC binding), similar to previous observations with NC [30,66] and other multivalent cations [67,68]. As the nature of NC/dsDNA binding is largely electrostatic, this NC-induced dsDNA softening is likely due to a transient localization of NC within dsDNA grooves, accompanied by local groove closure, or bending, around the protein [67,68]. Further increasing NC concentration leads to two sub-saturating regimes: partial dsDNA condensation and complete dsDNA condensation (Figure 3B,C). At concentrations around 10 nM NC (Figure 3C), we observe a phase-separation phenomenon never reported for dsDNA condensation by multivalent cations, but recently observed with NC [30,69] and protamine [70]. We observe saturating amounts of NC localizing and inducing globule formation in part of the DNA, while the rest of the dsDNA remains completely or almost completely protein-free and uncondensed (Figure 3C(iii)). This effect is also observed in force-extension measurements where the partial condensation force plateau spans only a fraction of the dsDNA length and the rest of the dsDNA stretches as expected for a protein-free molecule (Figure 3C(iv)). This partial plateau has an average condensation force lower than the maximum value achieved at saturating [NC], and gradually grows and spans more dsDNA length as [NC] increases.

At a critical concentration ([NC] = 20 nM in 50 mM Na^+^), the condensation force reaches a maximum ($F_{c,max}=9\pm 1\;pN$) and spans the whole dsDNA length (Figure 3D(iv)). In parallel AFM studies, the same concentration regime corresponds to the whole dsDNA substrate condensing into a tight spherical globule with no central hole, as illustrated in Figure 3D(i) and observed with AFM in Figure 3D(iii). At NC concentrations significantly exceeding optimal conditions, the condensation force first goes down (from ~10 pN at 20 nM NC to ~5 pN at 1 µM NC) and then disappears completely at about 10 µM NC (Figure 3E(iv)). The disappearance of the condensation force plateau corresponds to the absence of NC-induced dsDNA self-attraction, indicating dsDNA re-solubilization has occurred (Figure 3E(i)). This decondensed dsDNA becomes rigid as in the absence of bound protein, and cannot be observed on the AFM surface, presumably due to the NC/dsDNA complex undergoing a charge reversal, i.e., excessive NC binding that switches the net charge of the NC/dsDNA complex from net-negative to net-positive, such that it cannot attach to the positively charged Spd^4+^-treated mica surface.

### 3.2. Basic Residue HIV-1 NC Mutants Are Defective in dsDNA Binding and Condensation

**Figure 4:**
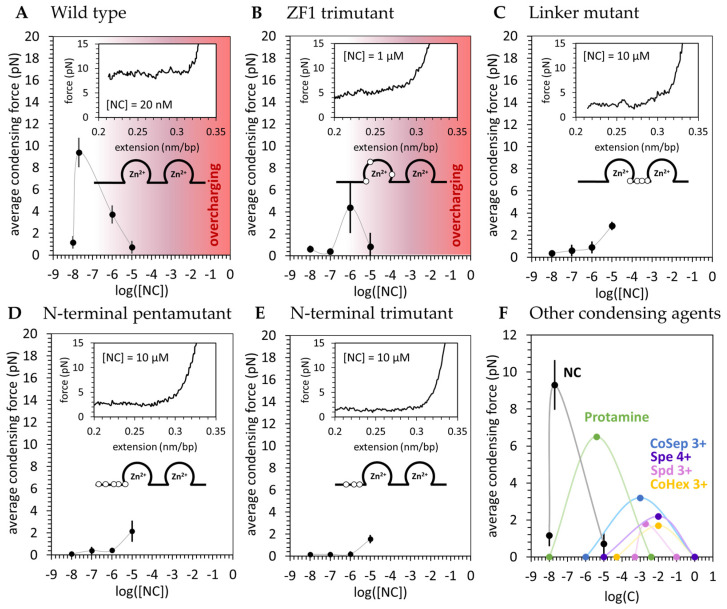
Basic residue mutations inhibit NC-induced dsDNA condensation. The average condensing force is plotted as a function of condensing agent concentration. A representative return trace is shown in each inset. The full set of experimental replicates can be found in Figure A1. (**A**) The average condensing force is plotted as a function of wild type HIV-1 nucleocapsid protein (WT NC) concentration. Error bars are standard error of the mean for *n* = 3 measurements. WT NC achieves a maximum average condensing force at [NC] = 20 nM. Above this critical concentration, the system becomes overcharged and condensing force decreases. (**B**) The zinc finger 1 (ZF1) trimutant variant achieves a maximum average condensing force at [NC] = 1 µM, ~50× higher than WT. (**C**–**E**) The N-terminal and linker basic residue variants only show condensation at [NC] = 10 µM. The maximum condensing force observed for these variants is lower than those observed for the ZF1 trimutant and the WT, and occurs at a 10× higher concentration. Overcharging is not observed for these variants at the concentrations studied. (**F**) The maximum average condensing forces and corresponding concentrations are reported for previously studied condensing agents, as detailed in Table A1. Data points show the minimum and maximum concentrations required for condensation and the maximum condensation force and corresponding concentration. Solid lines are provided to guide the eye and do not necessarily represent the exact distribution shape. NC achieves a maximum condensing force at a >100× lower concentration than reported for previously studied condensing agents.

**Figure 5:**
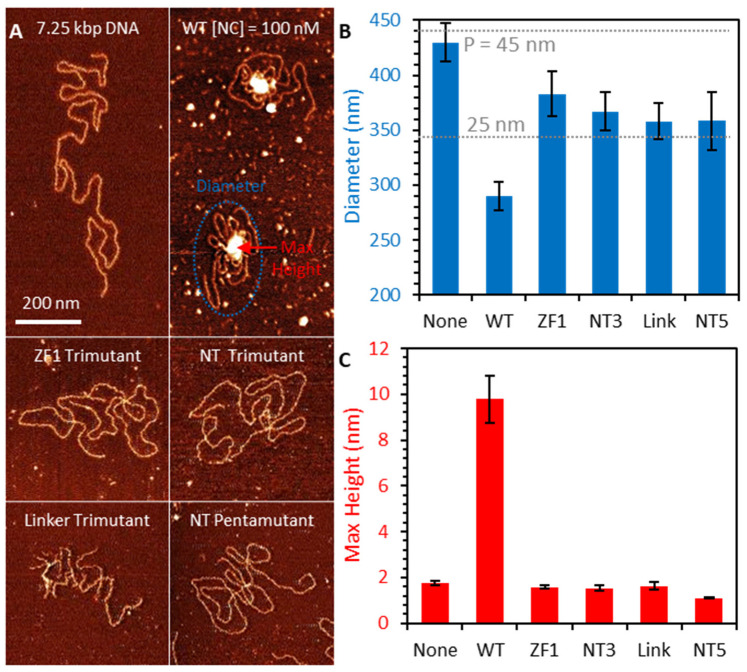
AFM imaging of dsDNA-NC complexes. (**A**) dsDNA in the absence of NC spreads out on the imaging surface, with minimal self-intersection. Incubation with 100 nM WT NC prior to deposition causes DNA to partially condense, both creating a large, multilayer globule with increased height (red) and reducing the spread of the DNA (blue). Incubation with NC mutants (also 100 nM) increases DNA flexibility, slightly reducing spread and increasing self-intersection, but does not create condensed globule. (**B**) Average diameter of DNA constructs incubated with each NC variant shows large reduction for WT NC, but a smaller effect consistent with a 2-fold reduction in DNA persistence length is observed for NC mutants. (**C**) Max height of DNA-NC complexes shows condensed globule formation for WT NC only, with NC mutants leaving DNA in single layer conformation.

The stretching experiment described in Section 2.2 was repeated for the four basic residue mutants of HIV-1 NC presented in Figure 1. The average condensing force $F_{c}$ (the height of the force plateau taken as the average force measured for extensions ≤ 0.3 nm/bp) is presented as a function of [NC] for both the WT and basic residue variants (Figure 4). $F_{c}$, or the free-energy of the system per unit length, is directly proportional to the strength of NC-mediated dsDNA condensation. WT NC achieves a maximum average condensing force of F_c_ = 9 ± 1pN at [NC] = 20 nM (Figure 4A). All the cationic NC mutants studied here required concentrations of at least two orders of magnitude higher than the WT to produce much a lower dsDNA condensation force (Figure 4B–E). AFM imaging of dsDNA-NC complexes showed consistent results, with the cationic NC mutants unable to condense DNA at the same concentration at which WT NC is effective (Figure 5. The NC variant with three cationic residues mutated in the first zinc finger (the ZF1 trimutant) was the least defective, showing only a ~twice lower condensation force plateau (F_c_ = 4.39 ± 2.33 pN) under optimal concentration conditions ([NC] = 1 mM) (Figure 4B). The most defective NC variants in both dsDNA binding and condensation were the penta- and tri-mutant N-terminal NC variants (Figure 4D,E), for which the condensation force was barely measurable and charge inversion was never observed. NC variants with three cationic residues mutated within the linker connecting its two ZFs were observed to be nearly as defective (Figure 4C). Interestingly, previous in vitro studies on the same variants also found the ZF1 mutant to be the least defective in single-round infectivity [57] (Table 1), possibly linking NC-induced dsDNA condensation and successful HIV-1 infection. The apparently different contributions from each of these subsets of cationic residues means that NC’s dsDNA condensing abilities rely on electrostatic contacts made by specific residues. The most important positive residues appear to be located in the flexible parts of NC, which can closely approach and screen dsDNA charges. The cationic residues located in NC’s structured ZFs, however, are more likely to be positioned further away from the dsDNA, and have a much smaller contribution [37]. These results suggest NC’s basic residues confer a significant part of the ability of NC to bind to and condense nucleic acids, particularly those features observed in common with dsDNA condensation by small multivalent cations.

**Figure A1:**
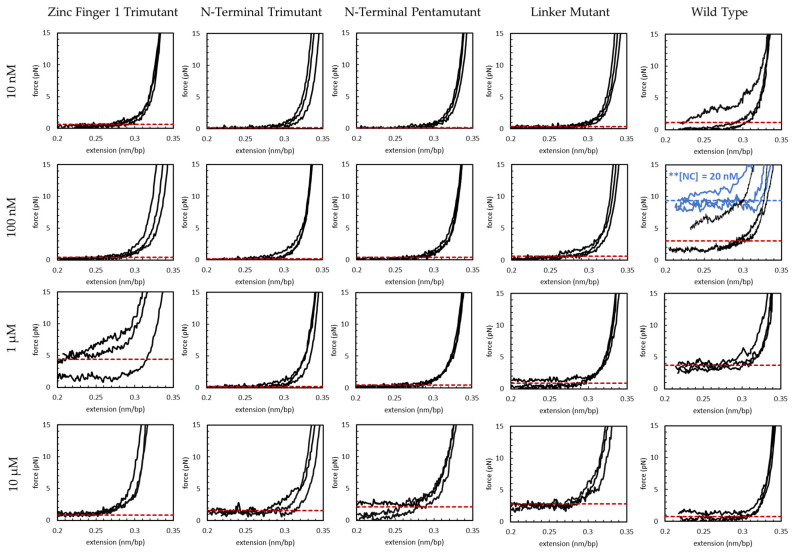
*n* = 3 replicates are shown for return curves of varying concentrations of WT and basic residue variants of HIV-1 NC. The average condensing force (measured below 0.3 nm/bp) is shown for each case (red dashed line). Data for ** [NC] = 20 nM is included in the 100 nM wild-type panel (blue solid lines) to illustrate the maximum average condensing force (blue dashed line) observed for WT NC.

AFM imaging of dsDNA-NC complexes showed consistent results, with the cationic NC mutants unable to condense DNA (produce a localized globule) at the same concentration at which WT NC is effective (Figure 5). However, increased dsDNA flexibility is still observed, as evidenced by increased dsDNA self-intersection. Additionally, the measured spread of the DNA on the surface (the minimum bounding diameter that contains the whole dsDNA molecule) is consistent with the NC mutants, causing a 2-fold decrease in dsDNA persistence length. Evidence of this persistence length decrease is also seen in OT experiments by small reductions in extension while held above condensing forces (Figure A1).

## 4. Discussion

### 4.1. NC Strongly Condenses DNA through a Counterion Correlation Mechanism That Is Sensitive to Cationic Mutations

Taken together, our results suggest HIV-1 NC condenses dsDNA in a way that is qualitatively similar to dsDNA collapse by small multivalent cations. Specifically, saturated binding of NC is required to condense dsDNA into its globular state. Based on previous studies [36], saturating conditions are reached when one NC is bound per ~5 bp. We expect this stoichiometry to correspond to the maximum NC-induced dsDNA condensation force, and to a 1:1 NC:DNA phosphate charge ratio within the complex—the critical limit at which charge neutrality is achieved. In our OT experiments, we found this point at 20 nM NC (in 50 mM Na^+^), corresponding to a maximum condensation force of 9 ± 1 pN. For an [NC] above or below this critical concentration, the condensation force decreases from its maximum. Below 10 nM NC and above 10 µM NC, the condensation force disappears completely. Qualitatively similar behavior has been observed for other multivalent cations, including Spe^4+^, Spd^3+^, CoHex^3+^ [50,51], Arginine (Arg)-rich proteins such as protamine [53,54,55,56], Lysine-rich H1 histone [53], polyArg, and polyLys [71], although higher concentrations of these cationic molecules were required to condense and re-solubilize dsDNA than was required for NC. For all previously studied cations, a minimal critical concentration c_min_ was required for cooperative condensation of dsDNA into a toroidal globule, and a maximum condensation strength was observed at c_mid_—the point at which complex charge neutrality is achieved. At yet higher concentrations, c_max_, a re-solubilization transition from globular to uncondensed dsDNA, was observed. Our results for WT HIV-1 NC are compared with previously published results for other condensing agents studied with OT in Figure 4F.

The phenomenon of dsDNA re-solubilization with increasing concentrations of multivalent cations is predicted to be accompanied by a complex charge inversion [47,48,49]. The switching of the dsDNA complex from net-negative (incomplete dsDNA screening by cations) to net-positive (dsDNA overcharging by cations) was measured previously as a switch in migration direction on gels with increasing concentrations of multivalent cation [53]. In this work, we observe the switch in the sign of the NC/dsDNA complex as the change in its ability to absorb onto a positively charged (Spe^4+^ treated mica) AFM surface. At low NC/dsDNA binding ratios, many complexes can be seen on the AFM surface. However, as [NC] increases, progressively fewer complexes attach to the surface, until eventually no complexes can be seen. This result, in combination with the disappearance of the condensing force at high concentration in OT measurements, leads us to conclude that excessive NC/dsDNA binding leads to a complex charge inversion, consistent with the counterion correlation mechanism of NC-induced dsDNA condensation.

Our OT experiments show the NC/dsDNA globule condenses along a constant force plateau, as previously observed for other multivalent cations [50,51,67]. However, the maximum NC-induced dsDNA condensation force is substantially higher than all those observed for dsDNA collapsed by multivalent cations (Figure 4F). The existence of the constant force plateau suggests NC does not condense dsDNA via multiple crosslinks, but rather by inducing a strong uniform dsDNA self-attraction [30]. This contrasts with our previous work [30], in which we thought NC might compact the DNA substrate via NC-stabilized dsDNA crosslinks in addition to the force of NC-induced dsDNA self-attraction. We previously reported large (>1 μM) dsDNA loops that were stable to high forces upon stretching, and attributed these structures to NC-mediated crosslinks [30]. However, our finding that cationic NC mutants are severely defective in dsDNA condensation leads us to believe that specific interactions involving NC’s zinc fingers do not play as large a role in dsDNA condensation as previously thought. Furthermore, similar “stick-slip” events have been observed for dsDNA treated by the multivalent cation spermine [72], which condenses dsDNA via a purely electrostatic interaction. We propose these high force “stick-slip” events upon DNA stretching (which we observe even after removing the free NC) to be the result of dsDNA tangling within the NC-condensed globular state as DNA relaxes to find its maximally compact state. Once free NC is removed (dissociated from DNA within ~1 min [30]), these extensive DNA entanglements remain and take much longer to disappear. It will be the work of future studies to clarify the nature of the dsDNA tangling mechanism and the time scale of its relaxation.

In another similarity to DNA collapse by multivalent cations, we find the position and number of positive charges on HIV-1 NC to have a critical effect on its condensing ability. Specifically, cationic NC mutants with different subsets of Arg or Lys residues mutated to neutral Ala (Figure 1) are defective in both dsDNA binding and condensation (Figure 4). The defects are more severe when charges are eliminated in NC’s flexible linkers, specifically in the N-terminus, resulting in a nearly 4-fold reduction in the condensation force. The effect of charged residue mutations within the first zinc finger structure were less pronounced, resulting in a two-fold reduction of the condensation force. Neither charge reversal nor dsDNA re-solubilization were observed for these NC mutants within the concentration range studied (<10 mM NC). We therefore conclude that the ability of NC to strongly condense DNA is conferred not by its charged residues alone, but also by their ability to be oriented as closely as possible to nearby dsDNA phosphates [37]. This is a property allowed in NC’s flexible protein domains (linker regions) and prohibited in its structured protein domains (ZFs). We hypothesize that these effects, in combination with the protein’s local charge density, contribute to the ability of NC to tightly bind and condense dsDNA.

All the aforementioned features of NC-induced dsDNA condensation are consistent with the counterion correlation mechanism [46,47,73], in which multivalent cations bind a negatively charged dsDNA surface in a primarily electrostatic mode. This strong but non-specific binding mode allows for each cation to remain delocalized when bound to the dsDNA, retaining the ability to move almost freely along the dsDNA surface. Since these cations have a large positive charge (≥+3), their high concentration on the dsDNA surface leads to a strong mutual repulsion [46]. This, in turn, leads to the development of a charge pattern on the overall neutral cation/dsDNA complex, where concentrated areas of positive charge alternate with locally unscreened areas of negative charge on the dsDNA surface (Figure 3D(ii)). As this charge pattern is mobile, it is free to adjust with respect to adjacent dsDNA surfaces to position excess plus charge against excess minus charge in counterphase, thereby minimizing the free energy of the system and leading to short-range electrostatic dsDNA self-attraction. The strength of dsDNA self-attraction will therefore be directly proportional to the charge, concentration, and mobility of the condensing counterions. This type of dsDNA condensation requires saturated cation/dsDNA binding, such that there are sufficient counterions bound per base pair to induce a strong mutual repulsion and position correlation. The condensation force plateau is the signature of this type of dsDNA condensation, arising from NC-induced force of dsDNA self-attraction. Upon stretching molecules condensed by NC, we observe large force spikes associated with the release of a significant length of dsDNA [30]. This force-extension behavior is characteristic of the breaking of protein-induced inter-dsDNA crosslinks, which rely on a non-electrostatic specific protein attachment to at least two distant sites on polymeric dsDNA. Surprisingly, we observe similar force spikes even after complete dissociation of NC from the condensed DNA globule. We hypothesize that these force spikes are not related to crosslinking by residual non-electrostatically bound NC molecules, but instead are the result of dsDNA entanglement that takes a long time to relax. Our next study will focus on elucidating the nature of this effect and characterizing the kinetics of dsDNA decondensation. Additionally, the noncovalent dsDNA crosslinking is not expected to exhibit a critical protein concentration required for the all-or-nothing state of NC-induced dsDNA condensation. Instead, protein-mediated dsDNA crosslinks can accumulate gradually upon protein binding, leading to a steady increase in protein/dsDNA density. However, in contrast to what is predicted by the counterion correlation mechanism and our observations, high concentrations of dsDNA crosslinking protein are not expected to lead to protein/dsDNA complex re-solubilization.

The hydration model is an alternative model for multivalent cation-induced dsDNA condensation [74,75,76]. Like the counterion correlation model, the hydration model also predicts a uniform dsDNA self-attraction induced by complementary charge patterns arranging their waters of hydration on opposing dsDNA surfaces. However, in contrast to the counterion correlation model, the hydration model of dsDNA condensation predicts a charge pattern to arise from cation absorption into periodic features of the dsDNA helix—such as its major and minor grooves—instead of mobile multivalent cations self-organizing via mutual repulsion on the dsDNA surface. For HIV-1 NC, this tight localization would be inconsistent with the high mobility measured for NC bound to duplex RNA [44] as well as with the relatively fast NC/dsDNA dissociation rate (~20 s^−1^) measured in our previous work [30]. Also, the hydration model of dsDNA attraction implies adjacent dsDNA helices are positioned parallel to each other, with their localized periodic charge patterns arranged in counterphase by a specific dsDNA orientation relative to each other. While it is possible to imagine that such an ordered parallel array of dsDNA molecules may exist within a toroidal DNA globule induced by small multivalent cations like Spd^3+^ or CoHx^3+^, the NC-induced dsDNA globule is not toroidal, and does not have long stretches of parallel DNA helices. Instead, dsDNA is curved and bent to maximize its density within the globule. Therefore, we do not believe the hydration model of NC-induced dsDNA self-attraction is consistent with our observations. It also does not explain the complex overcharging and dsDNA re-solubilization we observe at high NC concentrations. Some fraction of bound NC may be transiently localized within the structural elements of the B-DNA double helix, leading to the hydration attraction mode, while other NC molecules remain more mobile and correlated with each other, leading to the counterion correlation mechanism of dsDNA self-attraction. Further studies are required to determine the role of each type of NC-induced dsDNA self-attraction in the observed condensation phenomenon.

### 4.2. DNA Condensation by NC Is Unusual in Its Ability to Phase Separate on DNA and Induce a Spherical Globule Instead of a Toroid

While we observe many similarities in dsDNA condensation by NC and other multivalent cations, we also find significant differences. Most importantly, the force of NC-induced dsDNA self-attraction is much stronger than the condensing forces measured for all other studied multivalent cationic molecules (Figure 4F). As estimated above, the maximum per bp dsDNA self-attraction induced by NC ($G_{bp}\sim 0.8\;k_{B}T/bp=0.5\;Kcal/mol\cdot bp$) is several fold stronger than the dsDNA self-attraction induced by the four cationic NC mutants studied here and all previously studied multivalent cations. This is likely the reason why sub-saturating amounts of NC can phase-separate on dsDNA, leading to a fraction of the total dsDNA length being saturated with NC and condensed into a tight spherical globule while the rest remains protein-free and uncondensed (Figure 3C(iii)). Since NC partitioning on dsDNA requires a significant loss of entropy, this process must be driven by a sufficiently high free energy of NC-induced dsDNA condensation. This kind of globule/coil phase-separation within a single polymeric dsDNA molecule was never reported for other cationic condensing agents, which typically transition suddenly from completely uncondensed to completely condensed as the concentration of the condensing agent crosses above a critical value [33].

Another important distinction is that NC-induced dsDNA condensation leads to the formation of a dense spherical dsDNA globule (Figure 3C,D(iii)), while all other multivalent cations studied (except protamine [70]) were observed to form toroidal DNA condensates with a measurable central hole. This effect is likely the result of two NC properties: (i) the exceptionally strong NC-induced dsDNA self-attraction, reported by the height of the force plateau; and (ii) a significant increase in dsDNA flexibility, reported here as an observed reduction in the dsDNA persistence length. Theory [77] predicts that the combination of strong dsDNA self-attraction and high flexibility will lead to a space-filling spherical, rather than toroidal, globule in a sufficiently long dsDNA molecule. It was shown [77] that the critical length L*, above which dsDNA forms a spherical instead of toroidal globule depends on the strength of dsDNA self-attraction α, the dsDNA persistence length p, the base pair length b=0.34 nm, and the dsDNA diameter (including its hydration layer) d~3 nm, as follows:

(2) $$L*\sim {\left(\frac{bp}{\alpha }\right)}^{3}\frac{1}{d^{5}}$$

The above expression reflects the dependence of L* on the fundamental parameters of the condensing agent/dsDNA complex. This expression is correct to within a factor of 10, as it neglects the corresponding numerical coefficients. For NC-condensed dsDNA, α≈0.48 (in units of $k_{B}T$ per bp), p≈20 nm, and Equation (2) yields $L_{NC}^{*}\sim 10\;bp$. In other words, a dsDNA molecule longer than just a few dsDNA persistence lengths will form a spherical globule when exposed to sufficient NC. In contrast, dsDNA with an unperturbed persistence length (p≈50 nm) experiencing a ~4-fold weaker dsDNA self-attraction—as is in the case of Spe^4+^ (with α≈0.12)—is predicted to form a toroidal globule when shorter than $L_{Spe}^{*}\sim 10\;kbp$.

### 4.3. NC-Induced DNA Condensation Is Consistent with a Possible Function to Prevent Premature Capsid Uncoating during Reverse Transcription Leading to Defects in Viral Replication

We hypothesize that the ability of NC to collapse dsDNA into a dense spherical globule has biological relevance in preventing premature uncoating, which is initiated by the rupture of the HIV-1 capsid during endogenous proviral dsDNA synthesis. It was recently shown that reverse transcription of a ~10 kbp viral RNA genome into ~10 kbp of proviral dsDNA happens within the tiny free volume in the mature HIV-1 capsid, which is similar to the self-volume of the synthesized dsDNA [29]. Therefore, even when the high elasticity of the capsid [14] is considered, the extraordinarily strong dsDNA condensation by NC is critical in mitigating intra-capsid pressure and preventing premature uncoating. In a previous work [29], we estimated the interhelical DNA spacing in the NC-condensed spherical globule to be $d_{NC}\sim 3\;nm$, which is equivalent to the effective diameter of hydrated uncondensed dsDNA [78,79]. In other words, the net volume occupied by the proviral dsDNA and all its associated NC molecules (~1 NC per 5 bp) is the same as the volume of the uncondensed dsDNA alone. NC is a 55 aa long protein with a molecular weight of ~6.6 kDa. Its net volume in the condensate constitutes ~20% of the uncondensed dsDNA volume, and a significant fraction of all of the available intra-capsid volume. The “disappearance” of the NC volume is, therefore, expected to strongly reduce intra-capsid pressure, and may be sufficient to prevent premature capsid uncoating [29].

The inter-helical dsDNA distance estimated for the NC-induced globule ($d_{NC}\approx 3\;nm$) is only slightly larger than that measured for DNA condensed by the strongest known condensing agent Arg_6_ ($d_{Arg6}\approx 2.85\;nm$), similar to the distance measured for dsDNA condensed with Lys_3_Arg_3_ ($d_{Lys3,Arg3}\approx 3\;nm$), and smaller than the distance measured for Lys_6_-condensate dsDNA ($d_{Lys6}\approx 3.2\;nm$) [71]. However, as mentioned above, HIV-1 NC is not a 6 aa-long peptide, but rather a 55 aa-long protein with two structured zinc fingers (Figure 1), which contribute a significant self-volume. This means NC is an effectively stronger condensing agent than these cationic polypeptides, and able to bring dsDNA closer together by squeezing more water out of the condensate [50,51].

Taken together, our results suggest that HIV-1 NC is optimized to make it the one of the most effective DNA condensing agents observed with OT (Table A1). We show that it is not just the charge density that makes NC such an effective condensing agent, but also the location of the charges. In particular, positive charges located in more flexible regions of the protein are most important in conferring strong DNA self-attraction and condensation. Finally, we find that NC cationic mutations that strongly inhibit DNA condensation also exhibit significant defects in HIV-1 replication (Table 1). Although NC participates in many stages of viral replication, our results are consistent with the hypothesis that defects in DNA condensation contribute to defects in HIV-1 replication.

## Figures and Tables

**Table 1:** Infectivity properties of wild type nucleocapsid protein and basic residue variants as measured in cells [57].

Variant	RNA Packaging	Single-Round Infectivity	Relative Multiple round H9 Infectivity
Wild-type	100	100	1.0
N-terminal trimutant	51 ± 19	7.4 ±1.6	(3.8 ± 3.3) × 10^−3^
Zinc finger 1 trimutant	31 ± 14	8.8 ± 2.4	(5.0 ± 4.4) × 10^−5^
Zinc finger linker mutant	61 ± 11	3.2 ± 0.7	(7.2 ± 4.5) × 10^−5^
N-terminal pentamutant	3.0 ± 0.3	0.016 ± 0.007	≤(7.1) × 10^−6^

## Data Availability

The data presented in this study are available on request from the corresponding author.

## Appendix A

**Table A1:** Properties of various condensing agents. Reported values for the minimum (C_min_) and maximum (C_max_) concentration of condensing agent needed for observable condensation. The maximum condensing force (F_max_) is achieved at an intermediate concentration (C_mid_). The effective charge of each condenser (Z_eff_) and buffer conditions for previous studies are also displayed.

Ligand or Protein	C_min_ (M)	C_mid_ (M)	C_max_ (M)	F_c,max_ (pN)	Z	Z_eff_	Buffer
Spd ^3+^ [51]	5.0 × 10^−4^	2.0 × 10^−3^	1.0 × 10^−1^	1.80	3	3	10 mM Tris, pH 7
CoHex ^3+^ [50]	5.0 × 10^−5^	1.0 × 10^−2^	1.0 × 10^0^	1.70	3	3	10 mM Tris, pH 7
CoSep ^3+^ [50]	1.0 × 10^−6^	1.0 × 10^−3^	1.0 × 10^0^	3.20	3	3	10 mM Tris, 50 mM NaCl
Protamine [50]	1.0 × 10^−8^	4.0 × 10^−6^	4.0 × 10^−3^	6.50	50		10 mM Tris, 50 mM NaCl
Spe ^4+^ [50]	1.0 × 10^−5^	1.0 × 10^−2^	1.0 × 10^0^	2.20	4	4	10 mM Tris, 50 mM NaCl
NC ^3.5+^	1.0 × 10^−8^	2.0 × 10^−8^	1.0 × 10^−5^	9 +/− 1	11	3.5	10 mM Hepes, 45 mM NaCl, 5 mM NaOH, pH 7.5
